# Gated Skip-Connection Network with Adaptive Upsampling for Retinal Vessel Segmentation

**DOI:** 10.3390/s21186177

**Published:** 2021-09-15

**Authors:** Yun Jiang, Huixia Yao, Shengxin Tao, Jing Liang

**Affiliations:** College of Computer Science and Engineering, Northwest Normal University, Lanzhou 730070, China; jiangyun@nwnu.edu.cn (Y.J.); taoshengxin11@gmail.com (S.T.); 2020211969@nwnu.edu.cn (J.L.)

**Keywords:** deep convolutional neural work, retinal vessel segmentation, gating mechanism, skip-connection, adaptive upsampling

## Abstract

Segmentation of retinal vessels is a critical step for the diagnosis of some fundus diseases. ***Methods:*** To further enhance the performance of vessel segmentation, we propose a method based on a gated skip-connection network with adaptive upsampling (GSAU-Net). In GSAU-Net, a novel skip-connection with gating is first utilized in the extension path, which facilitates the flow of information from the encoder to the decoder. Specifically, we used the gated skip-connection between the encoder and decoder to gate the lower-level information from the encoder. In the decoding phase, we used an adaptive upsampling to replace the bilinear interpolation, which recovers feature maps from the decoder to obtain the pixelwise prediction. Finally, we validated our method on the DRIVE, CHASE, and STARE datasets. ***Results:*** The experimental results showed that our proposed method outperformed some existing methods, such as DeepVessel, AG-Net, and IterNet, in terms of accuracy, F-measure, and AUC${}_{R}{}_{O}{}_{C}$. The proposed method achieved a vessel segmentation F-measure of 83.13%, 81.40%, and 84.84% on the DRIVE, CHASE, and STARE datasets, respectively.

## 1. Introduction

According to the World Vision Report (https://www.who.int/publications/i/item/world-report-on-vision accessed on 5 September 2021) released by the World Health Organization in October 2019, more than 418 million people worldwide have glaucoma, diabetic retinopathy (DR), age-related macular degeneration (AMD), or other eye diseases that can cause blindness. Accurate segmentation of retinal images is an important prerequisite for doctors to perform a professional diagnosis and prediction of related diseases. In current clinical practice, however, manual visual inspection is usually employed to obtain this morphological information, which is laborious, time-consuming, and subjective [1]. Automatic segmentation algorithms can help doctors analyze complex fundus images, and the accuracy is gradually improving, which has attracted more attention in recent years. Currently, tremendous efforts have been made on retinal vessel segmentation. These methods can be broadly divided into four main categories: window-based processing methods, classification-based methods, tracking-based methods, and deep-learning-based methods.

Window-based processing methods: Chaudhuri et al. [2] used two-dimensional matched filters for retinal vessel segmentation. Based on this, a segmentation method that combines multiscale matched filtering and dual thresholding was proposed [3]. In [4], a segmentation algorithm based on the Gabor filter was proposed. In [5], a Cake filter was proposed, which can better detect elongated structures in images. The method based on window processing can maintain the original structure of blood vessels and has a better segmentation effect on thin blood vessels, but it needs to process each pixel, so it has the drawback of large computation and long time consumption.

Classification-based methods: The classification-based method first trains the classifier by extracting the feature vectors of the pixels and then classifies the segmentation regions obtained from the low-level processing into blood vessels or backgrounds. Some of the common classifiers are the KNN classifier proposed by Salem et al. [6] and the AdaBoost classifier proposed by Carmen et al. [7]. Classification-based methods generally require manual extraction of features and manual selection of classifiers. Therefore, there is still much space for improvement in the efficiency of the algorithm.

Tracking-based methods: The tracking-based method first determines an initial seed point, then from that point, iteratively following the characteristics of the vessel, such as vessel width, position direction, etc. The semi-automatic vascular tracking algorithm starts with the initial point and initial direction, using a width priority search, iteratively searching for vessels. A tracking algorithm for manually determining the initial point was proposed by Liu et al. [8], which achieved the final segmentation by continuously finding new starting points for resegmentation in the remaining vessels. In [9], some of the brightest points in the vascular pixels were found and used as starting points. In [10], a particle filter was used for retinal vessel tracking. Since semi-automatic vascular tracking algorithms rely on the determination of the starting point, they have been gradually replaced by fully automated vascular tracking algorithms [11,12]. The method based on fully automatic vascular tracking is very adaptive, but relies heavily on the selection of initial seed points and direction.

Deep-learning-based methods: Deep-learning-based approaches generally first build a training model using blood vessels and background data and then use the training model to classify each pixel in retinal images. A typical example based on convolutional neural networks (CNNs) was the blood vessel segmentation method proposed by Khalaf et al. [13]. The author used a CNN containing three convolution layers to perform blood vessel segmentation. Fu et al. [14] proposed a complete convolution network called DeepVessel. They used a side output layer to help the network learn multiscale features. In addition, the encoder and decoder structures are widely used in fundus image segmentation due to their excellent feature extraction capability, especially U-Net [15]. On the basis of the encoder and decoder, Wu et al. [16] proposed VesselNet based on a multiscale method. Feng et al. [17] proposed a cross-connected convolution neural network (CcNet) for blood vessel segmentation, which also adopted a multiscale method. In order to improve the segmentation ability of the network, the attention mechanism was gradually applied to retinal vessel segmentation. Zhang et al. [18] proposed an attention-guided network (AG-Net) for blood vessel segmentation.

Efforts have been made to improve the accuracy of retinal vessel segmentation. The basic network for blood vessel segmentation has been extensively developed; especially the network with the U-Net structure has become more and more popular. However, the skip-connection between the encoder and decoder in U-Net is too simple, resulting in noise being transmitted to the decoder as well. In order to restore the original image size for pixel-level prediction, upsampling in the decoder is usually realized by bilinear interpolation and deconvolution. A drawback of the oversimple bilinear interpolation is that it does not take into account the correlation between each pixel. These problems all lead to broken microvessels in the segmentation results of the model, low accuracy, and sensitivity of the model to noise and lesions.

To tackle the above problems, a gated skip-connection network with adaptive upsampling (GSAU-Net) is proposed to segment retinal vessels. The main work of this paper includes the following contents:

- We propose a gated skip-connection network with adaptive upsampling (GSAU-Net) to segment retinal vessels. A gating is introduced to the skip-connection between the encoder and decoder. A gated skip-connection was designed to facilitate the flow of information from the encoder to the decoder, which can effectively remove noise and help the decoder focus on processing the detailed information;
- A simple, yet effective upsampling module is used to recover feature maps from the decoder, which replaces the data-independent bilinear interpolation used extensively in previous methods. Compared to deconvolution, it improves the performance of the model with almost no additional computational cost;
- Finally, comprehensive experiments were performed to evaluate the proposed method on three public datasets (DRIVE [19], CHASE [20], and STARE [21]), showing its effectiveness.

The remainder of this article is organized as follows. The second section describes the proposed method in detail, including the network backbone structure, gated skip-connection, and adaptive upsampling. The third section introduces the datasets, experimental setting, and evaluation index. In the fourth section, our experimental results are discussed and compared. Finally, the conclusion is drawn in the fifth section.

## 2. Methods

In this section, we first introduce the network structure, then elaborate on the details of the modules in the network in detail.

### 2.1. GSAU-Net Architecture

**Figure 1:**
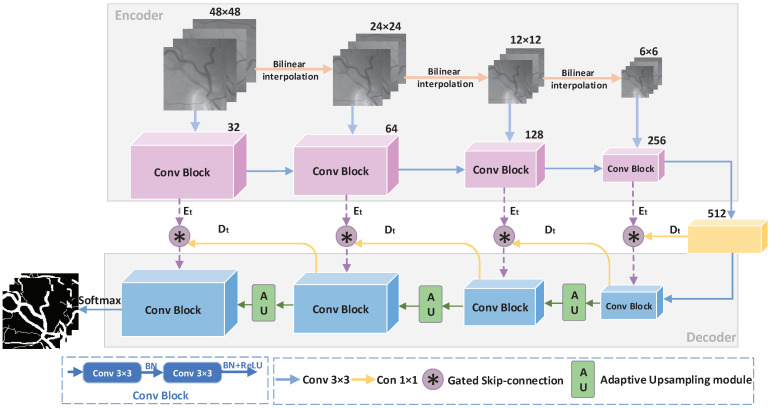
The GSAU-Net architecture.

In this section, we present our GSAU-Net architecture for semantic segmentation. As depicted in Figure 1, our network consists of a decoder and an encoder. The encoder part is the upper part of the figure with multiscale input added. It is used to extract the important semantic features of fundus images. The other part is the decoder at the bottom of the figure. Between the encoder and the decoder, we enforced a gated skip-connection to transmit only the information useful for restoring the original image to the decoder. At the same time, it can also eliminate background noise and transmit low-level semantic information to high-level semantic information. This can help the decoder focus on processing the relevant boundary-related and detailed information.

In the encoder, we give the original image with an input scale of 48 × 48, after a bilinear interpolation process to obtain three different scale images of 24 × 24, 12 × 12, and 6 × 6. Adding multiscale inputs to the encoder can ensure the network learns features at different scales, which can improve the robustness of the network. The encoder contains four downsampled convolution blocks. Each convolution block consists of two 3 × 3 convolution layers, and the convolution layers are regularized by a batch normalization layer. The end of the two convolution layers is followed by an activation layer, which is the rectified linear unit (ReLU) layer. In order to reduce the information loss caused by convolution and pooling in the downsampling, a 3 × 3 convolution is used in the downsample process.

In the decoder, four convolution blocks are used to restore the feature map reduced due to downsampling to the original image size. In the downsampling of the network, the small edge information of the image is weakened and blurred. To capture the correlation between adjacent pixels in the feature map, we used adaptive upsampling instead of bilinear interpolation and deconvolution for upsampling. Between each convolution block, adaptive upsampling could enlarge the feature map once, so that the feature map gradually approaches the original image size.

#### 2.1.1. Gated Skip-Connection

**Figure 2:**
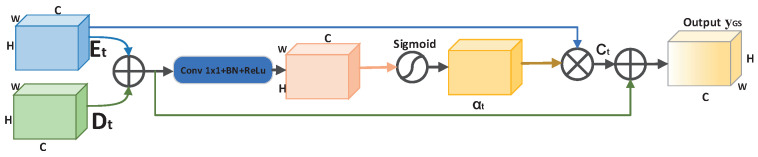
Structure of the gated skip-connection.

In U-Net [15], the encoder transmits information directly to the decoder by skipping the connection, and noise is also transmitted to the decoder in this process. Then, we introduce the gated skip-connection (GS) to reduce noise. The GS modifies the original skip-connection to serve as a bridge between the encoder and the decoder to transmit information. The structure of the GS is shown in Figure 2. In GSAU-Net, we use the GS to transmit the edge information of the encoder to the decoder, and low-level semantic information and high-level semantic information can also be transitioned through the GS.

We use the GS in multiple locations between the encoder and decoder. Let *t* denote the number of locations. *t* ∈ 0,1,2,3. *E*${}_{t}$ is the boundary semantic information transmitted from the encoder, and *D*${}_{t}$ is the information sampled by the decoder. To apply the GS, we first obtain an attention feature map by concatenating *E*${}_{t}$ and *D*${}_{t}$ followed by a 1 × 1 convolution layer (Conv${}_{1\times 1}$). This convolution layer is followed by a sigmoid function σ in turn. $\alpha_{t}$ is calculated from Equation (1).

(1) $$\alpha_{t}=\sigma \left({\mathrm{Conv}}_{1\times 1}\left(E_{t}⊕D_{t}\right)\right)$$

where ⊕ denotes the concatenation of feature maps. Given the attention map $\alpha_{t}$, GS is applied on *E*${}_{t}$ as an elementwise product ⊙ with $\alpha_{t}$ followed by a skip-connection. *C*${}_{t}$ is obtained by this operation. The result of the aggregation of *E*${}_{t}$ and *D*${}_{t}$ and *C*${}_{t}$ is processed by channelwise concatenation. At each pixel, the output of *Y*${}_{G}{}_{S}$ is computed as Formula (2).

(2) $$\begin{array}{cc} Y_{GS} & =C_{t}+\left(E_{t}⊕D_{t}\right)\\ \; & =\left(E_{t}⊙\alpha_{t}\right)+\left(E_{t}⊕D_{t}\right) \end{array}$$

Important information about the boundary region in the fundus image is obtained by the gated skip-connection processing of *E*${}_{t}$ and *D*${}_{t}$, that is the attention weight map about the boundary region.

#### 2.1.2. Adaptive Upsampling

**Bilinear interpolation:** Bilinear interpolation is often used to scale or enlarge images, and it is one of the most frequently used in upsampling. Mathematically, bilinear interpolation is a linear interpolation extension of the interpolation function with two variables. The core idea is to perform one linear interpolation in each of the two directions.

If the size of the source image is m × n and the target image is × b, then the side length ratios of the two images are m/a and n/b, respectively. Note that, usually, this ratio is not an integer and is programmed to be stored as the float type. The pixel value (*i*,*j*) of the target image can be expressed as (*i* × m/a, *j* × n/b). Obviously, this corresponding coordinate is not an integer in general, and noninteger coordinates cannot be used on discrete data such as images. Bilinear interpolation calculates the value of the pixel (grayscale or RGB value) by finding the four pixel points closest to this target coordinate. If the image is a grayscale image, then the grayscale value *f*(*i*,*j*) of the point (*i*,*j*) can be expressed by Equation (3).

(3) $$f\left(i,j\right)=w_{1}\times p_{1}+w_{2}\times p_{2}+w_{3}\times p_{3}+w_{4}\times p_{4}$$

where *p*${}_{i}$ (*i* = 1, 2, 3, 4) is the nearest four pixel points and *w*${}_{i}$ (*i* = 1, 2, 3, 4) is the corresponding weight value of each point. This method is computationally small, but does not take into account the effect of the rate of change of gray values among the neighboring points. This results in the loss of the high-frequency component of the scaled image, and the image edges become blurred to some extent.

**Deconvolution:** When using a neural network for pixel prediction, the size of the output often becomes smaller as the input image is extracted by a convolutional neural network (CNN). Sometimes, we need to restore the image in its original size for further computation (e.g., semantic segmentation of the image). This operation of mapping the image from small resolution to large resolution by expanding the image size is called upsampling. Deconvolution is generally used for upsampling. The deconvolution layer, to which people commonly refer, first appeared in Zeiler’s paper [22] as part of the deconvolutional network.

The following properties affect the output size *o* of a convolutional layer: *i* is the input size; *k* is the kernel size, *s* is the stride (the distance between two consecutive positions of the kernel); *p* is the zero padding (number of zeros concatenated at the beginning and at the end of an axis). When performing deconvolution, it can be broadly divided into the following two relationships.

Relationship 1 [23]: For (o+2p−k)%s=0,

(4) o=s(i−1)−2p+k

Relationship 2 [23]: For (o+2p−k)%s≠0,

(5) o=s(i−1)−2p+k+(o+2p−k)%s.

Compared to bilinear interpolation, deconvolution adds additional parameters and computational overhead to the model.

To further improve the performance of the network, adaptive upsampling is introduced to recover feature maps from the decoder. Adaptive upsampling is achieved by dividing the feature map into *r* × *r* subwindows and periodically rearranging the information in the subwindows, compressing the number of channels of the feature map and, thus, expanding the height and width to achieve the upsampling effect. The larger *r* is, the larger the feature map after upsampling. In this paper, *r* was set to 2, 4, and 6. Since adaptive upsampling learns the correlation between the feature map and the ground truth, the upsampled feature map contains more information. Adaptive upsampling has the following advantages as an upsampling method. First, compared with bilinear interpolation for upsampling, although simple and without introducing additional parameters, it is data-independent and cannot accurately restore the lost feature information. Second, compared to deconvolution, adaptive upsampling improves the performance of the model with almost no additional computational cost. It is learnable and data-dependent, so it can capture and recover more detailed information lost in downsampling than bilinear interpolation and adaptive upsampling. Adaptive upsampling is implemented as shown in Algorithm 1.
 **Algorithm 1:** Adaptive upsampling module.
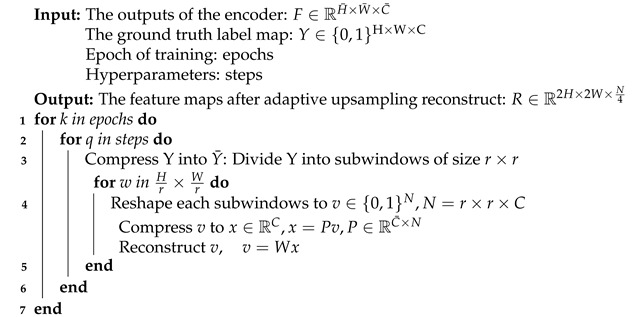


## 3. Datasets and Evaluation

### 3.1. Datasets

The proposed method was validated on three public datasets (DRIVE [19], CHASE [20], and STARE [21]). In the DRIVE dataset, there are 40 retinal images, corresponding to the ground truth images and mask images. The size of each image is 565 × 584 pixels. The first 20 images were used as the training set, and the last 20 images were used as the test set.

The CHASE dataset consists of 28 retinal images, corresponding to the ground truth images and mask images, each of which is 1280 × 960 pixels in size. For the CHASE dataset, we adopted the partition method proposed by Zhuang et al. [24], which divided the first 20 images into a dataset and the remaining 8 into a test set.

The STARE dataset contains 20 retinal images, corresponding to the real ground truth images and mask images. Each image is 700 × 605 pixels in size. We used the leave-one-out method to generate a training set and a test set. Each image was tested once. Finally, the final evaluation result was obtained by summing and averaging the evaluation indexes.

Since there are only a small number of sample sets to train the network structure, but the training of the deep neural network requires a large number of training samples, we expanded the training dataset by using the random patch method [25] on the image, which is very important to improve the accuracy of segmentation, prevent overfitting, and ensure the robustness of the network.

### 3.2. Experimental Environment and Parameter Settings

Our deep learning framework was implemented with the open-source package Pytorch. The server environment was Quadro RTX 6000. Ubuntu64 was the operating system. The method used for training was the random patch method of Jiang et al. [25]. This method was trained by extracting random patches of 48 × 48 pixels from the training set for the input of the network. The model was trained with a total of 200 epochs and a batch size of 256. Each image was generated with 10,480 patches for training. The Adam optimizer was used for model training, and the initial learning rate of the model was 0.001. In the parameters of the Adam optimizer, the exponential decay rate was the default value of 0.9. The step decay method was used to update the learning rate. The decay coefficient and the weight decay coefficient were set to 0.01 and 0.0005, respectively.

The loss function of the model is a cross-entropy loss function. It is expressed mathematically as follows:

(6) $${\mathrm{Loss}}_{ce}\left(y,\hat{y}\right)=-\sum y_{i}\log{\hat{y}}_{i}+\left(1-y_{i}\right)\log\left(1-{\hat{y}}_{i}\right)$$

where $y_{i}$ means the real label and ${\hat{y}}_{i}$ represents the predicted label.

### 3.3. Performance Evaluation Indicator

In this paper, by generating a confusion matrix, the sensitivity, specificity, accuracy, F-measure, and other evaluation indicators were calculated, and the performance of retinal image segmentation was analyzed. The calculation of each evaluation index is as follows:

(7) $$\mathrm{Accuracy}=\frac{TP+TN}{TP+FN+TN+FP}$$

(8) $$\;\mathrm{Sensitivity}\;=\frac{TP}{TP+FN}$$

(9) $$\;\mathrm{Specificity}\;=\frac{TN}{TN+FP}$$

(10) $$\;\mathrm{Precision}\;=\frac{TP}{TP+FP}$$

(11) $$F-\mathrm{measure}=\frac{2\times \;\mathrm{Precision}\;\times \;\mathrm{Sensitivity}}{\mathrm{Precision}+\;\mathrm{Sensitivity}}$$

Here, *TP* is the number of correctly divided blood vessel pixels, *TN* is the number of correctly divided background pixels, *FP* is the background pixel incorrectly divided into blood vessel pixels, and *FN* is the blood vessel pixel incorrectly marked as the background pixel.

## 4. Experiment Results and Analysis

### 4.1. Comparison of the Results before and after Model Improvement

To verify the effectiveness of our proposed GS module, other attention modules were also added to the baseline network, multi-scale Uet (MUet), for comparison with the GS module. We used the MUet network as a quantitative model and selected two typical attention modules, which can be embedded in other models to compare with our GS module. Among them, the first is the efficient channel attention (ECA) module of ECA-Net [26], which is often used in object detection and instance segmentation tasks. ECA is improved from the SE module [27]. It was empirically shown that avoiding dimensionality reduction and appropriate cross-channel interaction are important to learn effective channel attention. Another attention module is the dense atrous convolution module (DAC) of CENet [28], which uses the Inception structure and atrous convolution to capture more high-level information and preserve spatial information for 2D medical image segmentation. In this paper, the numbers in bold in the table represent the best results under the corresponding metrics.

Table 1 shows the experimental results of the GS module with the ECA module and DAC module added into the baseline network, MUet, respectively. Although two attention modules, ECA and DAC, improved the performance of the model to a certain extent, from the two evaluation indicators of accuracy and F1, the overall segmentation results of the GS were higher than those of the two attention modules embedded in the MUet network. Compared to the other two methods, the GS module has the smallest number of parameters. This is because the GS can facilitate the flow of information from the encoder to the decoder, which can effectively remove noise and help the decoder focus on processing the detailed information.

In Table 2, we compare the impact on model performance when upsampling using adaptive upsampling (AU), bilinear interpolation (Bilinear), and deconvolution (Deconv), respectively. As can be seen from Table 2, the number of parameters of the model is minimal when the decoder uses bilinear interpolation for upsampling. Since bilinear interpolation is data-independent and unlearnable, it cannot learn the relationship between pixels, and the model has the worst segmentation effect compared to the other approaches. Upsampling using deconvolution adds additional parameters and computational cost while improving model performance. The adaptive upsampling proposed in this paper has a better segmentation effect than deconvolution without increasing the number of parameters. AU can recover the image more closely to the original, compared to Bilinear and Deconv.

### 4.2. Evaluation of ROC and Precision Recall Curves before and after Model Improvement

**Figure 3:**
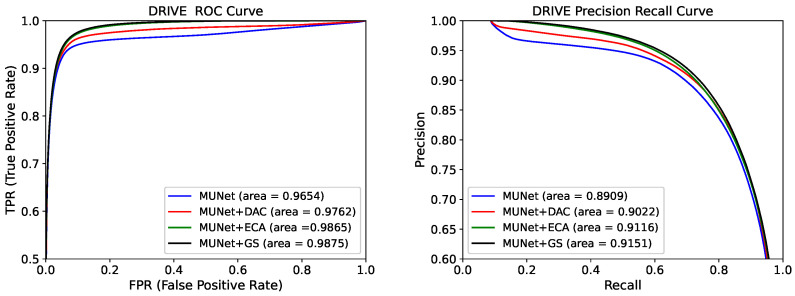
Receiver operating characteristic (ROC) curve and precision recall (PR) curve for five models on the DRIVE dataset.

**Figure 4:**
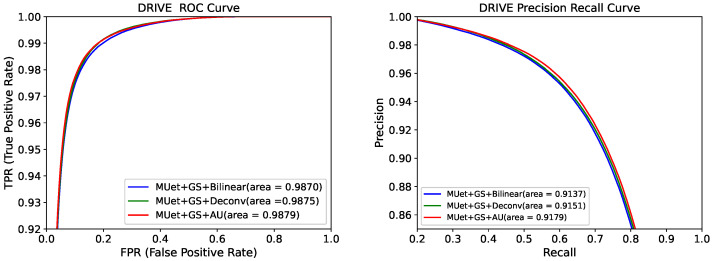
Receiver operating characteristic (ROC) curve and precision recall (PR) curve for five models on the CHASE dataset.

In Figure 3 and Figure 4, we compare the ROC and PR curves of different attention modules and upsampling methods, respectively. The closer the ROC curve is to the upper left corner, the higher the accuracy of the model. The point on the ROC curve closest to the upper left corner is the best threshold with the least number of classification errors and the lowest total number of false-positive and false-negative cases.

It can be seen from Figure 3 that the ROC and PR areas of the network containing the GS module were the largest. This was due to the gating and skip-connection joined in the GS module, which further filtered and reduced noise from the encoder. As shown in Figure 4, the ROC and PR areas of the network containing the AU module were larger than other upsampling methods. This is because the AU module reduces the loss of the network in upsampling compared to bilinear interpolation and deconvolution. This further showed that the GS and AU modules had better performance than other attention and upsampling modules.

### 4.3. Comparison of Segmentation Results with Different Methods

In order to further verify the effectiveness of the proposed algorithm for retinal vessel segmentation, the proposed method was compared with some existing methods on the sensitivity, specificity, accuracy, and F-measure on the three datasets of DRIVE, CHASE, and STARE. Table 3, Table 4 and Table 5 show the experimental results of different methods on the DRIVE, CHASE, and STARE datasets, respectively.

For the DRIVE dataset, the F-measure of retinal vessel segmentation for this method reached 83.13%, which was 1.71% higher than U-Net [31]. GSAU-Net uses the gated skip-connection to filter background noise, which can distinguish the pathological region very well. We used adaptive upsampling instead of bilinear interpolation and deconvolution. This can alleviate the difficulty of upsampling to restore tiny thin vessels so that the segmentation results are more accurate. However, the highest accuracy was shown by D-Net.

For the STARE dataset, we used the leave-one-out method for training and testing, and the best F-measure was 0.8976. The worst F-measure was 0.7832. The average segmentation results on the STARE dataset are shown in Table 5.

Although the sensitivity of U-Net [31] on the CHASE dataset was higher than that of our method, the segmentation effect on small blood vessels was not very good, and sometimes fractures occurred. Moreover, our method had the highest F-measure; the specificity remained relatively stable; the noise contained in the segmented image was relatively small.

In Table 3, Table 4 and Table 5, we compare the proposed method with previously proposed methods, such as D-Net [32], MRA-Net [34], and MFI-Net [36]. The evaluation metric results of our methods were superior on the CHASE dataset. In D-Net [32], parallel convolution layers with different dilation rates are used to obtain more dense feature information. In MRA-Net [34], the residual attention and the spatial activation module are used to improve the feature extraction capability of the network. In MFI-Net [36], a fully aggregated skip-connection alleviates information isolation between the shallow and deep layers of the network. The segmentation time of D-Net, MRA-Net, MFI-Net, and GSAU-Net was 1.5 s, 5.96 s, 0.86 s, and 0.75 s for one image on the DRIVE dataset, respectively. Since the proposed model is a lightweight network, the final segmentation for fundus retinal images was also faster. This is friendly for clinical diagnosis, but also due to the lightweight nature of the model, there are limitations for image feature extraction compared to D-Net, MRA-Net, and MFI-Net.

### 4.4. Visualization Results

**Figure 5:**
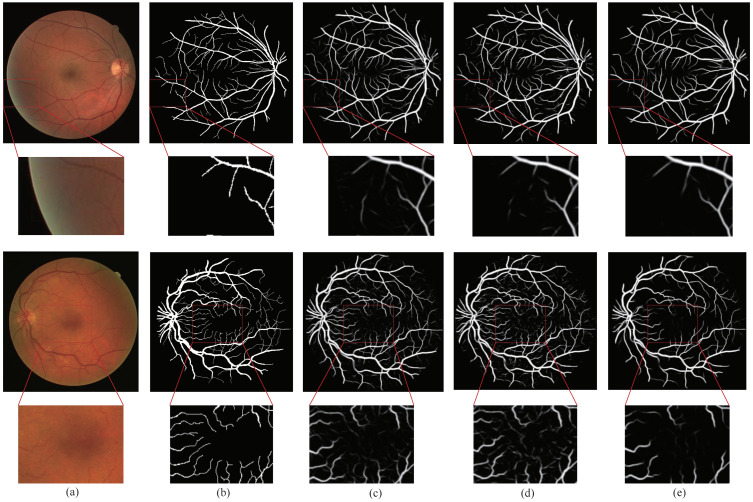
Comparison of the attention module visualization results on the DRIVE dataset. (**a**) Image; (**b**) ground truth; (**c**) ECA-Net [26]; (**d**) CE-Net [28]; (**e**) our method.

We compared the method proposed in this paper with the methods ECA-Net [26] and CE-Net [28]. Figure 5 is the visualization results of the DRIVE dataset. In Figure 5, Column (a) represents the original image, Column (b) represents the ground truth corresponding to the original image, Column (c) represents the segmentation result of ECA-Net [26], Column (d) represents the segmentation result of CE-Net [28], and Column (e) represents the segmentation result of the method proposed by us. The retinal vessels segmented by CE-Net [28] contained more noise, and the background was mistakenly segmented into blood vessels.

There were some problems such as unclear segmentation of small blood vessels at the edges and fuzzy boundaries. Although the retinal vessels segmented by ECA-Net [26] contained less noise, there were still some problems such as fuzzy boundaries and unclear small blood vessels. Compared with ECA-Net [26] and CE-Net [28], GSAU-Net can filter out more noise thanks to the GS module, and the AU module reduced the gap between the recovered image and the original image in upsampling. These made the network obtain more information about the tiny vessels. The background region noise of the fundus image segmented by our model GSAU-Net was the least. This also demonstrated the relevance and effectiveness of the GS module in background denoising.

**Figure 6:**
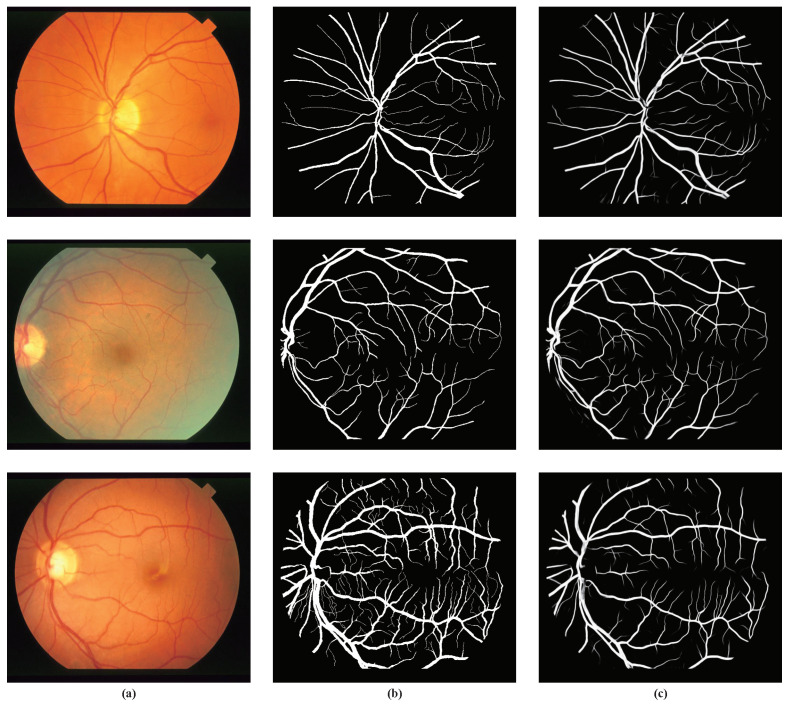
Experimental outputs for the STARE dataset using our method (**a**) Image; (**b**) ground truth; (**c**) our method.

The experimental outputs of our method on the STARE dataset are shown in Figure 6. It can be seen from the figure that the small blood vessels and noise areas were well segmented. However, the segmentation of the tiny blood vessels in the third picture still needs to be improved.

## 5. Conclusions

In this paper, we proposed a model named gated skip-connection network with adaptive upsampling (GSAU-Net) to segment retinal vessels automatically. In this model, a novel skip-connection with gating and adaptive upsampling was introduced to improve the traditional U-Net. In the extension path of U-Net, the gated skip-connection is utilized to facilitate the flow of information from the encoder to the decoder, which can effectively remove noise and help the decoder focus on processing the relevant boundary-related information. Due to some detailed information being difficult to recover, adaptive upsampling was employed. This could capture the correlation information between feature maps to improve the recovery performance of small vessels. Then, the feature maps are scaled to the same size as the input image, so as to achieve the pixelwise prediction. Finally, our method was verified on the DRIVE, CHASE, and STARE datasets. The experiment results showed that our method has better performance for retinal vessel segmentation compared to existing methods including DeepVessel [14], AG-Net [18], and IterNet [39].

Our model is lightweight due to the small number of hyperparameters in the gated skip-connection and adaptive upsampling. At the same time, the model is limited in its feature extraction capability for fundus retinal images, which leads to the need to improve the accuracy of the model. To improve the accuracy of the model, the future work will be to introduce the idea of generative adversarial networks and use the model in this paper as a generator. How to design an efficient discriminator for segmenting retinal vessels is also a necessary task.

## Figures and Tables

**Table 1:** Comparison of the proposed methods with other methods on the DRIVE dataset.

Method	Accuracy	Sensitivity	Specificity	F-Measure	AUC_*ROC*_	Params Size (MB)
MU-Net	0.9686	0.7345	0.9911	0.8040	0.9654	44.49
MU-Net + DAC [28]	0.9700	0.7770	0.9885	0.8195	0.9762	270.39
MU-Net + ECA [26]	0.9700	0.8116	0.9852	0.8258	0.9865	52.48
MU-Net + GS (Ours)	0.9701	0.8299	0.9836	0.8294	0.9875	47.98

**Table 2:** Comparison of the proposed methods with other methods on the DRIVE dataset.

Method	Accuracy	Sensitivity	Specificity	F-Measure	AUC_*ROC*_	Params Size (MB)
MU-Net + GS + Bilinear	0.9706	0.8115	0.9859	0.8290	0.9870	41.72
MU-Net + GS + Deconv	0.9701	0.8299	0.9836	0.8294	0.9875	47.98
MU-Net + GS + AU (Ours)	0.9706	0.8264	0.9845	0.8313	0.9879	47.97

**Table 3:** Comparison of the proposed methods with other methods on the DRIVE dataset.

Methods	Year	Accuracy	Sensitivity	Specificity	F-Measure	AUC_*ROC*_
FABC [7]	2010	0.9597	-	-	-	-
Cheng [29]	2014	0.9474	0.7252	0.9798	-	0.9648
Khalaf [13]	2016	0.9456	0.8397	0.9562	-	-
DeepVessel [14]	2016	0.9523	0.7603	-	-	-
Mo [30]	2017	0.9521	0.7779	0.9780	-	0.9782
U-Net [31]	2018	0.9531	0.7537	0.9820	0.8142	0.9755
Residual U-Net [31]	2018	0.9553	0.7726	0.9820	0.8149	0.9779
AG-Net [18]	2019	0.9692	0.8100	0.9848	-	0.9856
D-Net [32]	2019	0.9709	0.7839	0.9890	0.8246	0.9864
Lv [33]	2020	0.9558	0.7854	0.9810	0.8216	0.9682
MRA-Net [34]	2020	0.9698	0.8353	0.9828	0.8293	0.9873
SA-Net [35]	2021	0.9569	0.8252	0.9764	0.8289	0.9822
MFI-Net [36]	2021	0.9705	0.8325	0.9838	0.8318	-
Ours	2021	0.9706	0.8264	0.9845	0.8313	0.9879

**Table 4:** Comparison of proposed methods with other methods on the CHASE dataset.

Methods	Year	Accuracy	Sensitivity	Specificity	F-Measure	AUC_*ROC*_
Azzopardi [37]	2015	0.9563	0.7716	0.9701	-	0.9497
Deepvessel [14]	2016	0.9489	0.7412	-	-	-
U-Net [31]	2018	0.9578	0.8288	0.9701	0.7783	0.9772
Recurrent U-Net [31]	2018	0.9622	0.7459	0.9836	0.7810	0.9803
R2U-Net [31]	2018	0.9634	0.7756	0.9820	0.7928	0.9815
AG-Net [18]	2019	0.9743	0.8186	0.9848	-	0.9863
D-Net [32]	2019	0.9721	0.7839	0.9894	0.8062	0.9866
Lv [33]	2020	0.9608	-	-	0.7892	0.9865
MRA-Net [34]	2020	0.9758	0.8324	0.9854	0.8127	0.9899
MFI-Net [36]	2021	0.9762	0.8309	0.9860	0.8150	-
Ours	2021	0.9765	0.8170	0.9872	0.8140	0.9903

**Table 5:** Comparison of proposed methods with other methods on the STARE dataset.

Methods	Year	Accuracy	Sensitivity	Specificity	F-Measure	AUC_*ROC*_
Azzopardi [37]	2015	0.9497	0.7716	0.9701	-	0.9497
Miao et al. [38]	2015	0.9532	0.7298	0.9831	-	-
DeepVessel [14]	2016	0.9489	0.7130	-	-	-
Mo et al. [30]	2017	0.9674	0.8147	0.9844	-	0.9885
U-Net [31]	2018	0.9690	0.8270	0.9842	0.8373	0.9898
IterNet [39]	2019	0.9701	0.7715	0.9886	0.8146	0.9881
D-Net [32]	2019	0.9781	0.8249	0.9904	0.8492	0.9927
Lv [33]	2020	0.9640	-	-	0.8142	0.9719
MRA-Net [34]	2020	0.9763	0.8422	0.9873	0.8422	0.9918
MFI-Net [36]	2021	0.9766	0.8619	0.9859	0.8483	-
Ours	2021	0.9771	0.8535	0.9872	0.8484	0.9923

## Data Availability

We used three public datasets to evaluate the proposed segmentation network, namely DRIVE [19], CHASE [20], and STARE [21].
